# Supplementary material for: Are carbon emissions trading and green financial instruments synergistic? -Comprehensive quantitative research based on content analysis
Source: PLoS One. 2024 Mar 7;19(3):e0298601. doi: 10.1371/journal.pone.0298601 (PMC10919601; doi:10.1371/journal.pone.0298601)
Supplement: S1 Data — (ZIP) [file pone.0298601.s001.zip › Data/地级市Z_F工作报告.zip!/╦─┤¿/╥╦▒÷╩╨/└┤╫╘╣1⁄2╓┌║┼í╛┬φ┐╦╩2╛▌═°í┐_2014.docx]

各位代表：

我代表市人民政府，向大会报告过去一年的政府工作，对今年工作提出建议，请各位代表审查，并请市政协委员提出意见。

2013年工作回顾

2013年，国际国内发展环境复杂多变，我市经济发展遭遇建市以来的最大挑战，白酒、煤炭等支柱产业面临前所未有的发展困难。我们在省委、省政府和市委的坚强领导下，在市人大及其常委会的工作监督、法律监督以及市政协的民主监督下，围绕在次级突破战略布局中率先崛起、先于全省全面建成小康社会的奋斗目标，主动应对极为错综复杂的经济形势，努力克服地震等自然灾害的影响，创新思路、举措和办法，在艰难中拼搏、在逆境中奋起，奋力稳增长、调结构、抓改革、惠民生、促和谐，全市经济社会保持平稳较快发展，为率先崛起、全面建成小康打下了良好基础。

——过去的一年，我们努力克服经济下行压力，经济发展取得新成效。坚持稳中求进、止滑稳位，始终坚定发展信心不动摇，直面困难综合施策，着力助推经济增长。预计全年地区生产总值1345亿元，增长8%，总量保持全省第四位；规模以上工业增加值610亿元，增长6.5%，总量保持全省第三位；地方公共财政收入突破百亿元，达100.19亿元，增长23.31%。

——过去的一年，我们积极推动产业结构调整，转型升级迈出新步伐。坚持科学发展、创新驱动，依托市场倒逼产业转型，着力提升发展质量。预计全年完成技改投资222.3亿元，增长15.2%，单位生产总值能耗下降3.5%，服务业占比提高1个百分点。关闭了63个小煤矿。完成14户企业淘汰落后产能任务。成功创建国家农业科技园区。

——过去的一年，我们全面推进区域协调联动，县域经济凸显新亮点。坚持因地制宜、分类指导，落实多点多极支撑发展战略，创新实行差别化考核方式，加大以市带县力度，着力夯实底部基础。临港开发区升格为国家级经济技术开发区。翠屏区连续5年入围四川县域经济“十强县”，并跃居第六位，保持川南第一位。珙县、江安县、高县、长宁县经济总量可望突破百亿元。

——过去的一年，我们切实加快城乡统筹发展，城市农村呈现新面貌。坚持规划先行、城乡一体，加快推进新型城镇化，着力筑牢发展支撑。中心城区辐射吸纳和承载能力进一步增强。新农村建设成效显著，农村生产生活水平明显提升。预计全市新增城镇建成区面积12平方公里，城镇化率提高2个百分点，达43%。

——过去的一年，我们务实有力推进环境兴市，政风行风焕发新气象。坚持以身作则、强力实施，围绕服务态度好、办事效率高、发展成本低、干部队伍廉、社会环境优，着力打造一流环境。首次开通“企业家服务热线电话”，市级部门主要负责人在电视、报纸上公开承诺，组织开展政风行风评议，发展环境明显改善。

——过去的一年，我们全面加强民生政府建设，人民生活得到新改善。坚持依靠人民、造福人民，加快民生改善，凝聚发展正能量，着力促进社会和谐。预计城镇居民人均可支配收入22570元，增长10%；农民人均纯收入8780元，增长13%。“4·25”双河地震抗震救灾取得重大胜利，灾后重建有序推进。

一年来，我们以党的十八大精神为统领，认真落实市委四届六次全会的部署和要求，集中精力抓了以下六个方面的工作。

一、大力实施项目推进战略，筑牢经济发展支撑

坚持以项目建设稳增长、促转型，积极实施一大批大项目、好项目，预计全市全社会固定资产投资完成935亿元，增长25%。着力完善项目推进体制机制，成立29个项目指挥部，全方位推进重大项目建设。全年组织4次项目集中开工，开工项目385个、总投资714.1亿元。向家坝水电站蓄水至380米水位，6台发电机组投运；四川长宁—威远国家级页岩气示范区长宁区块建成6个水平井组，四川长宁天然气开发公司挂牌成立，标志着4200平方公里的长宁区块页岩气开发正式拉开序幕；筠连矿区新维、船景煤矿和溪洛渡—浙西特高压输变电工程等项目加快建设。宜宾至叙永高速公路建设稳步实施，成都至贵阳铁路宜宾段开工建设，宜宾机场迁建项目试验段工程、宜宾至昭通高速公路、绕城高速公路即将开工；内江至自贡至宜宾铁路、宜宾至西昌铁路和宜宾至毕节高速公路、宜宾至攀枝花高速公路前期工作进展顺利；宜宾至长宁、沙河至巡场公路改造全面完成；宜宾至屏山快速通道、筠连至巡司一级公路建设顺利推进；宜宾至庆符快速公路、南溪至阳春坝一级公路等项目前期工作进展顺利；宜宾港志城作业区重大件泊位即将开工建设，散货泊位完成可研报告。向家坝灌区一期工程前期工作顺利推进，灌区项目法人机构正式成立；王家沟、新坝、东山、龙滚滩、蟠龙湖水库等重点水利工程加快建设。新建、改建通村公路1100公里。储备未来五年固定资产投资项目2000余个，总投资超过6500亿元。

二、加快工业强市产业兴市，着力壮大经济规模

在稳增长中突出稳工业，围绕白酒、煤矿等重点行业和重点企业抓创新、调结构、促销售，工业发展速度实现止滑回升。着力帮助和引导白酒行业渡过难关，促进酒类企业推出新产品和创新营销模式，五粮液品牌价值701.58亿元，高洲、叙府、红楼梦等企业保持快速发展势头。全面完成煤矿企业兼并重组任务，比全省提前两个月复工复产。丝丽雅、天原、中核建中、惊雷科技经营效益较快增长。宜宾纸业整体搬迁、富源发电等技改项目有序推进。新增国家高新技术企业12家、国家级企业技术中心1家。13个重点园区累计入驻企业572户，预计园区规模以上工业总产值1180亿元，增长7.6%。临港开发区规划建设顺利推进，“飞地园区”取得实质性进展。五粮液园区列入国家级新型工业化示范基地，罗龙、阳春等4个园区列入全省“51025”重点发展产业园区，长宁县工业集中区等4个园区成功创建为全省循环经济示范区。强化中小企业培育，全市净增规模以上工业企业48户。加快农业农村发展，粮食稳定增产，茶叶、蚕茧产量居全省第二位，酿酒专用粮、畜禽、林竹、果蔬等特色优势产业进一步培育壮大。新建现代农业万亩示范区26个。农业产业化龙头企业发展到377户、农民合作组织2312个、家庭农场63个。初步建成新村536个、新农村综合体22个、幸福美丽新村39个，美丽乡村建设取得明显成效。推进服务业发展提质增速，预计服务业增加值增长11%，社会消费品零售总额495亿元，增长14%。“扩内需、促消费”系列活动有效开展，成功举办白酒文化节、早茶节等活动。财富中心、莱茵春天、西南轻工博览城等商业综合体建成运营。我市被列为四川省首批区域现代商贸物流中心和现代物流试点示范市。五粮液物流中心开工建设，宜宾港保税物流中心（B型）、“龙泉无水港”等项目有序推进。宜宾港集装箱吞吐量实现6.04万标箱，增长121%。高起点编制《宜宾市旅游发展总体规划》，注重市县（区）联动、梯次开发，加快建设国际生态文化旅游目的地，预计旅游业总收入206.6亿元，增长24.5%。交通银行、成都银行入驻宜宾，成都农商行发起设立的两家村镇银行顺利开业，五粮液集团财务公司获得筹建批复。全市农村信用社加快改制为农村商业银行。市商业银行改革发展进一步推进，科技支行正式成立。新设立小额贷款公司及融资性担保公司5家。预计全市银行信贷净投放140亿元，增长35.9%；银行存贷比达50.8%，提高5.6个百分点。保险、咨询、中介、电子商务加快发展。

三、科学有序开展城市规划，提升城镇建管水平

实施城市规划大会战，城市总体规划修编即将通过省政府批复，成为我市建市以来第一部经省政府批复的总体规划。环长江旅游景观大道规划方案等12个规划通过专家评审或规委会审查，县城及镇乡规划编制有序推进。临港新区、三江口区域、南溪滨江新城、宜宾县城北新区等区域加快建设，环长江旅游景观大道、三江口、滨江景观等一批提升城市形象的重点项目开工建设。中心城区旧城改造和风貌提升工程全面展开，城市道路黑化即将开工建设，三江河道得到有效治理。加快推进城市交通缓堵工程，继续抓好“公园、公交、公厕”和“停车场、洗车场、农贸市场”建设管理，强力推进城市公交改革，城市功能不断增强。推动建筑业及房地产业健康发展，预计建筑业总产值增长20%，房地产开发总投资增长34%。县城和重点镇加快发展。按照“属地管理、重心下移”的原则，加快城管体制机制改革，城市管理数字化、网格化、精细化水平明显提升。有序推进国家卫生城市和省级园林城市创建工作，城市美化、绿化、亮化工作成效明显。

四、坚定不移加大改革力度，增强开放合作实效

深化行政管理体制改革，扩权强县、扩权强镇改革试点有序推进。积极推进户籍制度改革，城市落户条件进一步放宽。大力实施农村综合配套改革，农村土地承包经营权确权登记工作全面推进，集体林权改革不断深化。推进财税体制改革，财政预算保障充分、执行有力，政府性债务风险保持在可控范围内。加快实施国有企业改革，统筹推进事业单位分类改革和教育、科技、文化、旅游等行业改革。深入推进医药卫生体制改革，21家县级公立医院取消药品加成。非公有制经济加快发展，预计实现增加值780亿元，占地区生产总值的比重达58%。招商引资工作取得新突破，全年共履约市外项目774个，实现市外到位资金632.48亿元，增长24.02%。积极参加西博会、华商大会、央企入川、知名民企入川、港澳企业入川等活动，取得丰硕成果。加强与长三角、珠三角等区域合作，推进川南城市群一体化建设，与德阳、绵阳等市州签订了战略合作协议。对外经贸合作继续拓展，引导特色产品扩大出口，茶叶出口占全省的80%以上，外贸进出口总额8亿美元，利用外资4549万美元。外事、台侨、贸促、海关、检验检疫等工作进一步加强。

五、统筹兼顾发展社会事业，提供优质公共服务

加快推进教育大市向教育强市转变，教育资源布局不断优化。计划三年投入25.9亿元，有针对性地解决农村义务教育突出问题，农村寄宿制学校等四大工程925个项目建设加快推进。义务教育均衡发展成绩显著，翠屏区、南溪区通过省上督导评估验收。高考本科上线人数连续8年增长。学前教育普及率有效提高。职教攻坚计划深入实施，宜宾卫校、宜宾商职校新校区建成投用。民办教育科学有序发展，宜宾天立国际学校竣工投用。宜宾学院、宜宾职业技术学院办学实力不断增强。文化惠民活动蓬勃开展，文艺创作成果丰硕。市广电中心、市档案馆主体工程完工。建成7个省级和10个市级示范乡镇综合文化站、22个市级示范农家书屋、55个留守学生之家、300个社区书屋、40个公共电子阅报屏，完成29万户家庭数字电视整体转换。新增五粮液老窖池遗址等全国重点文物保护单位7处，非物质文化遗产展览馆正式对外开放。严格实施国家基本药物制度，在全省率先实现省级定点医疗机构新农合即时结报。法定传染病总体发病率保持全省最低水平。市中医院门诊大楼全面完工，市二医院临港医院（儿童病区）、市妇幼保健院等项目有序推进。计生惠民工程全面完成，低生育水平持续稳定，被列入全国创建幸福家庭试点市。科技创新驱动示范、成果转化等五大科技工程项目加快实施。新增省级工程技术研究中心2家，获国家、省级科技立项71项，第三次蝉联全国科技进步先进市。群众体育活动蓬勃开展，圆满完成全运会女篮预赛等赛事承办工作，全省第十四届少数民族传统体育运动会筹备工作顺利推进。争创国家创新城市、全国质量强市示范城市和省级文明城市取得良好进展，工商管理进一步加强，食品药品机构改革圆满完成。新闻出版、统计、电子政务、地方志、住房公积金等工作成效明显，民族宗教、爱国卫生、红十字会、科协、工会、青少年、妇女儿童、老龄、残疾人事业取得新成绩。

六、坚持不懈加快民生改善，保持社会和谐稳定

投入专项民生工程资金90.22亿元，“十项民生工程”涉及的124个指标全面完成。积极创建省级创业型城市，多渠道促进高校毕业生等重点群体就业，预计全市城镇新增就业 6.3万人，城镇登记失业率3.7%。发放小额担保贷款0.96亿元、青年创业贷款1.3亿元。社会保险待遇稳步提高。超额完成养老、失业、医疗、工伤、生育保险扩面任务，城乡低保实现及时提标、应保尽保。市殡仪馆建成投用，市社会福利院迁建项目有序推进。扎实抓好保障性安居工程建设，新开工9022套，基本建成14764套，发放廉租住房租赁补贴11715户，“农民工住房保障行动”公共租赁住房分配入住1977套（间），改造农村危旧房7895户。9个集中连片扶贫开发和63个贫困村整村推进项目全面启动，全市共扶持贫困人口10.2万人。大力实施向家坝移民后续扶持发展工程，新增后期扶持向家坝农村移民23497人。物价总水平保持基本稳定。扎实推进治污减排，全市6大干法水泥厂和珙县电厂降氮脱硝工程建成投运，18户工业企业和13户规模化畜禽养殖企业污染限期治理任务全面完成。环境空气质量新标准自动监测系统（含PM2.5）建成投运。中心城区空气质量保持良好，二氧化硫、二氧化氮、可吸入颗粒物年均浓度值达到国家二级标准。长江出境断面水质达到Ⅲ类水域标准。森林覆盖率达42.61%。生态市建设加快推进，我市被省政府正式授予“四川省环境保护模范城市”称号，被联合国环境规划基金会等单位评为中国“杰出绿色生态城市”。革命老区、少数民族地区加快发展。积极开展对口援建，支援甘孜州雅江县和新龙县经济社会发展项目18个、投资4100万元。加强和创新社会治理，平安宜宾、法治宜宾建设扎实推进。第九届村（居）民委员会换届选举有序实施。成功创建为全国青少年权益工作创新试点城市。努力发挥“市长与网民”等平台重要作用，群众和信访工作进一步加强。狠抓安全生产管理和监督，未发生重大安全事故，全市生产经营性事故起数和死亡人数较大幅度下降。气象、防震减灾、公共危机预防和应急管理体系建设全面加强。积极支持驻宜部队建设，国防教育、人民防空、消防、征兵、优抚、安置、交通战备等工作成效明显，国防后备力量建设以及国家安全、保密工作取得新成绩。

各位代表，过去的一年值得我们总结和铭记。我们真切地体会到，在“人口多、基础弱、差距大、不平衡”的市情下推动科学发展、加快发展，必须力争“快”，始终坚持科学定位、高点起步、跨越赶超、奋勇争先；必须务求“好”，始终坚持结构调整、转型升级、统筹兼顾、提质增效；必须突出“新”，始终坚持解放思想、与时俱进、创新驱动、克难攻坚；必须立足“干”，始终坚持实干兴市、锐意进取、艰苦奋斗、敢于担当。

我们深深知道，过去一年各项成绩的取得，是全市上下在市委的正确领导下，同心同德、团结拼搏的结果，也是方方面面关心支持的结果。在此，我代表市人民政府，向各位人大代表、政协委员，并通过你们向全市各族人民、驻宜部队、武警官兵、政法干警、各民主党派、工商联、无党派人士、各人民团体和各界朋友表示衷心的感谢，并致以崇高的敬意！

同时，我们也清醒地看到，由于全国经济持续下行的影响与我市经济长期积累的结构性矛盾叠加交织，使去年我市经济发展一直处于特别困难的时期，特别是占工业经济比重近60%的白酒、煤炭两个行业受到较大冲击，影响了全市经济预期目标的实现；受规划编制、要素保障等因素的影响，一些城市项目没能迅速开工建设；现代农业和服务业比重还比较低，经济向好发展的基础还不牢固；城乡居民持续增收难度较大，保障和改善民生的任务依然繁重。我们高度重视这些问题，将在今后的工作中认真加以解决。

2014年工作安排

2014年是全面贯彻落实党的十八届三中全会精神、全面深化改革的第一年，是为实现率先崛起、全面建成小康社会奠定坚实基础的重要一年。纵观国际国内形势，我们仍处于可以大有作为的重要战略机遇期，具备许多有利条件和积极因素。党的十八届三中全会召开后全面深化改革将释放更多的制度红利，国家加快丝绸之路经济带、长江经济支撑带等区域发展和省上推进页岩气开发、建设重装基地等战略部署，为我市带来新的发展机遇。我们必须进一步增强进取意识、机遇意识、创新意识和责任意识，始终坚持发展第一要务，按照市委四届七次全会的工作部署，从最难处着眼，向最好处努力，牢牢把握工作主动权，开创跨越赶超的新局面。

今年政府工作的总体要求是：认真贯彻党的十八大和十八届二中、三中全会精神，全面落实中央、省委经济工作和城镇化工作会议部署，紧扣“两大目标”、构建“三个中心”、打造“六大基地”、实施“五大战略”，牢牢把握“稳位求效、创新求进”的工作要求，全面深化改革，加快转型发展，切实提高经济发展质量和效益，进一步保障和改善民生，促进全市经济持续健康发展、社会和谐稳定，为率先崛起、全面建成小康打下坚实的基础。

今年全市经济社会发展的主要预期目标是：全市地区生产总值增长10%以上；规模以上工业增加值增长11%以上；服务业增加值增长11%以上；全社会固定资产投资增长16%以上；社会消费品零售总额增长12.5%以上；地方公共财政收入增长12%以上；城镇化率提高2个百分点以上；城镇新增就业5.5万人，城镇登记失业率控制在4.5%以内；城镇居民人均可支配收入增长10%以上；农民人均纯收入增长12%以上；人口自然增长率控制在4.5‰以内；完成省上下达的物价调控、节能减排等目标任务。

上述指标安排，既充分考虑了发展大势和国家政策导向，又做到了与市委四届六次全会和“十二五”规划确定的目标相衔接。和全国、全省一样，预期增长速度相比往年有所调低，但对还处在调整转型期的宜宾来说，任务依然十分艰巨，需要我们加倍努力。我们将突出抓好八个方面的工作。

一、深化改革开放，增强发展动力活力

加快推进重点领域改革。进一步处理好政府与市场的关系，推动市场在资源配置中起决定性作用，更好地发挥政府作用，激发各类市场主体的活力和创造力。深化财税体制改革，实施全面规范、公开透明的预算制度。推进投融资平台公司可持续发展，严格控制政府性债务增量、逐步消化存量。放宽投资准入，鼓励和扩大民间投资。积极推进国有资产管理体制改革。分类制定市属国有企业改革方案，建立完善现代企业制度。大力发展非公有制经济。全面推进股份制改革，鼓励发展非公有资本控股的混合所有制企业。推进统筹城乡综合配套改革，全面深化农村产权、城乡社会保障、户籍、用地、农村金融创新等五项核心制度改革。深化粮食流通体制改革。深入推进事业单位分类改革。深化文化体制机制改革，完善文化市场准入和退出机制。推进自然资源及其产品价格改革。

切实打好临港开放开发攻坚战。突出发挥临港开发区工业经济转型发展的引领作用，加快建设产业港、开放港、创新港。坚持集群发展、联动发展、生态低碳发展，加快发展先进装备制造业和港口物流业，构筑现代港口产业体系。突出对内对外开放，进一步加强与上海港和沿江沿海发达地区的交流合作，强化产业链招商，建设招商引资和承接产业转移的新高地。加快打造航运中心，建设全域宜宾港，提升港口口岸功能，完善港口集疏运体系，确保宜宾港集装箱吞吐量突破10万标箱。着力简政放权、先行先试，加快临港开发区体制机制改革，推进与国家级开发区相适应的治理体系和治理能力现代化。

着力加快县域经济发展。加大沿江地区开放开发力度，着力建设沿江产业群、城镇群，不断增强辐射吸纳能力。充分发挥山区资源富集配套的优势，推进资源精深加工、就地转化和直接富民，培育发展特色优势产业。进一步完善差别化考核办法。加快推进扩权强县、扩权强镇改革。支持和鼓励各县（区）打破行政地域界限，发展“飞地”经济，实现协作发展、共同发展。加快革命老区、少数民族地区、贫困地区经济发展。

全面提升招商引资和对外开放水平。积极参加“中外知名企业四川行”和第十五届西博会等重大活动，加快引进国际国内大公司、大企业、大财团入驻或来宜建设生产、研发基地。大力推进与丝绸之路经济带、长江经济支撑带、成渝经济区、攀西资源开发试验区等区域内城市的交流合作，主动对接上海自贸区开放合作；力争实现招商引资市外到位资金700亿元。继续支持五粮液、丝丽雅等重点外贸企业加大出口，加强出口潜力企业的培育和扶持。大力吸引外资入驻，加快推进船景煤矿、嘉士伯啤酒等项目建设。加快对外劳务合作服务平台建设，鼓励有意向的企业到境外投资兴业。进一步做好外事、台侨、贸促、海关、检验检疫等工作。

二、加大投资力度，夯实发展后劲

深入实施项目推进战略。进一步健全指挥部责任制、全程督促检查制度、定期考核通报制度、风险责任金制度等项目推进机制，改善投资管理和服务，确保重大项目顺利实施。建立“要素资源跟着项目走”的保障机制，进一步创新投融资体制，努力向上争、着力市场融、突出对外引、协调银行贷、鼓励社会投，多渠道筹措项目建设资金。积极争取更多用地计划总量，加强节约集约用地，努力提高土地利用效率。切实完善重点项目审批“绿色通道”制度，促进项目尽快落地。适时组织实施重大项目集中开工。

切实加强重大项目建设。围绕全省通江达海交通枢纽中心建设，深入开展“立体交通大会战”，确保交通基础设施投资100亿元以上。强力推进宜宾至叙永、宜宾至昭通、宜宾至毕节、绕城高速等4条高速公路建设，推动宜宾机场迁建项目、成都至贵阳铁路宜宾段加快建设，力争开工建设内自宜城际铁路、宜宾港散货码头，推进长江上游宜宾段航道等级提升工作。加快宜宾至西昌铁路和宜宾至攀枝花、宜宾至仁寿、南溪经富顺至内江等高速公路前期工作；加大国省干道改造力度，全面推进农村公路建设。突出抓好技改扩能，确保工业投资400亿元以上。推动五粮液9万吨陶坛陈酿酒库、中核建中核燃料元件生产线、宜宾纸业整体搬迁等技改项目竣工投运。加快推进威力化工搬迁、金川电子磁性材料元器件、长虹红星电子搬迁等技改项目建设，切实开展珙县陶瓷生产线、筠连煤矸石电厂、长宁通用航空产业园项目前期工作。加快完善现代水利体系，组织实施好王家沟、东山、蟠龙湖、新坝、龙滚滩、二龙滩等水库和烟水重点水源工程建设。切实推进向家坝灌区工程前期工作。

强化项目储备包装。突出抓好产业招商和园区招商。围绕打造“六大基地”，依托特色资源和主导产业，加大项目策划包装、推介、对接工作力度，集中力量引进一批重大产业项目，带动配套企业跟进和中小企业发展。主动对接国家政策支持方向，做好保障性安居工程、城市基础设施、“三农”、交通基础设施、节能环保等领域的项目谋划、争取和推进工作。对项目储备库滚动充实、分类管理，加快推进前期工作，不断提高项目成熟度。

三、着力提质增效，增创工业新优势

改造提升传统优势产业。支持白酒企业加大产品结构调整力度，强化品牌酒和“宜宾酒”市场营销，加快筹建“宜宾名优白酒产业联盟”，巩固“中国白酒金三角”龙头和核心区地位。加快推进煤炭技改矿井和新建矿井建设，新增煤炭生产能力700万吨以上。进一步彰显资源特色，促进能源、矿产、农业等资源与技术、资本、人才相融合，催生以页岩气开发等为代表的大项目、大产业。继续实施大企业大集团培育计划，加大中小微企业培育力度，新增规模以上工业企业50户以上。

积极发展战略性新兴产业。推进工业化信息化深度融合，完善战略性新兴产业规划，全力推进新能源、新材料、高端装备制造、节能环保等产业加快发展。加快实施一批重点科技创新和改造项目，推进科技成果转化运用，力争技术创新投资增长20%以上、新创省级以上工业品牌15个以上。推动建立新材料、高端装备制造产业联盟，争取将惊雷科技产业园、国家精密模具与特种材料集成制造高新技术产业化基地等建设成为战略性新兴产业示范基地。

加快产业园区建设。完善全市产业园区一体化管理体制，推进统筹规划、分类指导、错位发展。推动老工业基地调整改造，加速“退城入园”，加快产业集聚、集群、集约发展。不断强化产业配套、生产保障和生活服务功能，力争园区基础设施投入60亿元。实施本土企业在外产业“回引工程”，积极支持跨区域合作。

四、强化城乡统筹，加快新型城镇化进程

加快完善城乡发展规划。切实编制好历史文化名城保护规划和城镇近期建设规划。积极推进岷江新区、临港新区、柏溪组团、罗龙组团、金沙新区、南溪新区等区域控规修编，抓好中心城区交通设施、教育设施、医疗设施、环卫设施、城市色彩、城市慢行系统、城市天际线控制、公共开敞空间等方面的专项规划编制。完成省、市级历史文化名镇名村保护规划编制。扎实推进建制镇规划编制，尽快实现村镇规划全覆盖。开工建设城市规划馆，加强规划成果的公示、展示。

切实加强城市建设管理。以新的城市总体规划为依据，全面提速城市建设，力争完成城乡建设投资360亿元以上，新增建成区面积20平方公里以上。围绕三江六岸六组团布局，大力推进南部新区、岷江新区、临港新区、南溪新区、金沙新区、李庄新区等区域开发建设。实施旧州组团南北区整体打造，加快建设三江口景观和环长江旅游景观大道。加快推进中心城区“五线三街”改造提升工程建设，加快实施城市道路黑化和人行道升级改造，大力推进地下管网、农贸市场、停车场建设。推动县城扩容提质，支持有条件的县发展成为中小城市。加快打造市级以上小城镇建设试点示范镇。着力提升城市品质，加快创建国家卫生城市和省级园林城市，强力推进城市管理规划、交通、标识标牌、园林绿化、户外广告、江河岸线等十大专项治理，推动城市面貌在上半年明显改观。

加快推进城乡一体化发展。积极推动县改区、乡改镇，进一步深化户籍管理制度和土地管理制度改革，稳步提高户籍人口城镇化水平。着力推进城乡规划、基础设施、公共服务一体化，促进城乡要素平等交换和公共资源均衡配置，形成以工促农、以城带乡、工农互惠、城乡一体的新型工农、城乡关系。

五、突出农民增收，促进农业和农村经济加快发展

构建多元化农民增收格局。继续强化农民增收工作县（区）委书记、县（区）长负责制，建立促农增收长效机制。加快发展特色优势产业，强化家庭经营性收入产业支撑。加强农民工职业技能培训和组织化输出，提高工资性收入水平。全面落实强农惠农富农政策，稳步提高社会保障水平，增加财产性收入和转移性收入。

大力发展现代农业。加快推进农业转型升级、提质增效，建设生态农业文明。推进农业规模化，加快土地流转和适度规模经营，切实保障粮食安全和主要农产品供给，集中连片发展优质粮油、茶叶、畜牧、林竹、果蔬、烤烟、蚕桑、水产等特色优势产业。建成120万亩造纸原料林基地。切实发展向家坝灌区现代立体农业，创建一批国家级、省级现代农业产业化示范基地。推进农业标准化，加强农产品质量安全保障体系建设。推进农业品牌化，加快“万亩林亿元钱”示范区建设，进一步提升“宜宾早茶”、“川红”、“南溪豆腐干”等公共品牌，加快建设世界红茶文化中心、中国茶业博览城和宜宾茶国际会展中心。大力发展特色优势农产品精深加工。新增市级以上龙头企业15户、年营销额500万元以上农民合作组织30个、家庭农场30个。推进农产品专业市场和营销网络体系建设，大力发展食品冷链物流。加强农产品经纪人培训。强化农业科技创新推广，推进新品种选育、新技术研发、新工艺改进、新产品开发。加强耕地质量建设。提高农作物耕、种、收综合机械化水平。

全面推进新农村建设。坚持产村相融发展，推动新村带产业、产业促新村。加快推进3个省级新农村示范县、8个市级示范片建设，确保完成三年规划目标任务的75%以上。高标准推进幸福美丽新村建设，沿高速公路和交通干线实施村庄整治，全面配套基础设施和公共服务设施，建成新村416个，其中幸福美丽新村21个。深入推广“春风模式”，加快推进第二轮1236个“千村春风工程”示范村建设，积极探索新农村综合体发展模式。加强农村公共服务，力争在省市新农村示范片、新农村综合体率先实现均等化。创新体制机制，让农民群众全面参与新农村规划建设。积极探索适合平坝、浅丘、深丘、山区等不同类区特点的新农村建设新路子。

六、扩大市场消费，推进服务业大发展

加快长江上游商贸物流中心建设。深入开展“扩内需、促消费”系列活动，推动农产品进城、工业品下乡，促进便利消费、节庆消费、热点消费和循环消费。大力实施《宜宾建设长江上游商贸物流中心规划》。推动上力理想城、东方时代广场等商业综合体全面建成投运。加快西南机械装备贸易城等项目建设，推动重庆新世纪百货等项目落地建设。支持沃尔玛、华润万家、大润发等商贸企业发展，引导叙府商城、燕君连锁、绿源食品等企业加快发展。积极推行连锁经营、电子商务等现代流通方式。切实推进五粮液物流中心和临港、象鼻、罗龙等物流园区发展，加快建设中国酒都粮食物流中心，规划建设空港物流中心。引导安吉、天畅等物流企业转型发展，培育发展第三方物流企业，积极引进大型物流企业。加快推进宜宾港保税物流中心（B型）、国家进口粮食指定口岸建设。

加快川南城市群金融中心建设。完善支持金融发展的政策，优化金融生态环境，深化政银企合作。鼓励创新金融产品和金融服务，拓展投融资渠道，努力增加信贷投入，降低融资成本，支持实体经济发展。鼓励企业直接融资，进一步推动企业上市，积极推进企业在成都（川藏）股权交易中心挂牌。积极推进市商业银行增资扩股和跨区域发展。力争在全省率先全辖完成农村信用社改制为农村商业银行。推动五粮液集团财务公司尽快开业。积极引进各类金融机构来宜设立分支机构，切实做好新设小额贷款公司和融资性担保公司增资扩股工作。大力推进市场信用体系建设。积极防范和化解金融风险。

加快国际文化旅游目的地建设。深入推进经营性文化单位改革，加快“6+2”重点文化产业发展，积极培育文化龙头企业。加快西南桃园影视城、南溪文化创意动漫产业园等重点项目建设，推进文化产业园区建设。切实办好早茶节、白酒文化节等活动。加快编制《宜宾市旅游总体规划》，创新旅游管理体制机制，促进旅游业发展转型升级。加强旅游基础设施、公共服务体系建设，切实打造长江国际生态欢乐海等项目。着力开发一批复合型旅游项目，大力发展度假休闲旅游，强化市场营销，力争实现旅游业总收入240亿元。

促进房地产业及其它服务业健康发展。切实加强住房保障，增加普通商品住房供应。整合检验检测资源，组建公共技术服务平台。加强规划引导，放宽市场准入，积极发展社区服务、家庭服务等民生性服务业，加快信息、咨询、中介等服务业发展。

七、建设生态文明，推动可持续发展

强力推进节能减排。严格控制“两高一资”和产能过剩行业新上项目。加快淘汰落后产能，优化用能结构。加快实施节能改造、节能技术产业化示范等重点工程。大力实施污染物总量减排，强化污染减排考核、监测、统计和预警调控体系建设。优化产业结构和布局，按时关停黄桷庄电厂，加快建设垃圾环保发电厂。

加大环境保护力度。全面启动创建国家环保模范城市工作。健全环境应急管理体系和辐射环境监管体系。加强规划和建设项目环境保护管理。加大城市环保基础设施建设力度，提高城市污水处理率，加强流域污染综合整治，确保饮用水源安全。制定和实施大气污染防治行动计划。深入推进机动车尾气、油烟、噪声污染、农村面源污染、土壤环境保护和综合治理工作。开展环保专项整治行动，切实加大环保执法力度。严守生态和耕地保护红线，坚持差别化供地政策，提高土地利用集约化水平。加强地质灾害防治、矿产资源勘查及开发利用。

加强生态文明建设。建立健全生态文明目标考核体系，着力推进绿色发展、循环发展、低碳发展，加快构建长江上游国际生态山水园林城市。完成《宜宾市生态市建设规划》修编和《宜宾市生态城市建设规划》编制。加强生态乡镇、生态村等“生态细胞”建设和农村环境连片整治工作。加大向家坝库区生态治理力度，大力实施《五粮液产业园区周边及岷江宜宾段生态环境保护规划》。巩固扩大天然林保护、退耕还林成果，完成造林25万亩，新增森林面积10万亩，森林覆盖率达到43%以上。加强老君山国家级自然保护区建设。大力推进城乡绿化，增强生态产品生产能力。

八、增进民生福祉，全面加强社会建设

切实保障和改善民生。继续实施“十项民生工程”，集中公共财力和资源抓好民生大事。实施更加积极的就业政策，做好以高校毕业生为重点的青年就业工作和农村转移劳动力、城镇困难人员、退役军人就业工作，促进创业带动就业。着力完善社会保障体系，加快社会保险市级统筹，实施“五险合一”统一征缴。推进城乡居民社会养老保险与企业职工基本养老制度并轨。构建和完善最低生活保障、特困人员供养等救助体系基本框架。大力发展慈善、互助、红十字会等事业，健全残疾人社会保障和服务体系。完成省政府下达我市的保障性住房建设任务。建立健全住房保障准入、退出和管理机制，确保分配公平、管理规范、群众满意。切实抓好市场价格监测管理，保持物价总水平基本稳定。基本完成“4.25”双河地震灾后重建。扎实抓好扶贫移民工作，扶持贫困人口6.5万人以上。实施好乌蒙山片区区域发展与扶贫攻坚规划。全面完成向家坝水电站库区剩余及新增影响区移民安置。

提升教育卫生发展水平。调整优化教育资源布局，大力抓好农村寄宿制学校等基础设施建设，改善农村义务教育学校办学条件。加快公办幼儿园建设，提高学前教育水平。继续深化课程和教学、招生考试制度以及义务教育质量评价等改革，深入推进教育信息化建设。实施教师素质提升三年行动计划，努力培育造就一批名教师、名校长、学科领军人才。切实保障进城务工人员子女等群体受教育的权利。探索高中阶段公办学校横向集团化办学。加快规划建设教育园区。支持宜宾学院向应用科技大学转型发展，加快宜宾职业技术学院发展。鼓励社会力量兴办教育。继续深化医药卫生体制改革，巩固完善新型农村合作医疗制度，全面实施国家基本药物制度，健全卫生应急管理和预案体系，加强基层卫生机构建设。加快推进市中医院住院部、市二医院临港医院（儿童病区）、市妇幼保健院迁建等项目建设，依托市一医院规划建设现代化医疗康健园。加强重点传染病防控工作。

扎实推进文化强市建设。加快构建覆盖城乡、惠及全民的基本公共文化服务体系。大力弘扬民族精神和时代精神，提升文艺创作水平，深化群众性精神文明创建活动。进一步繁荣哲学社会科学。完成市广电中心、市档案馆建设，力争开工建设市博物馆新馆和市群众文化体育活动中心，加快市文化馆项目前期工作。加快基层文化设施示范建设，推进全民阅读活动。加快乡村学校少年宫建设，实现全覆盖。基本完成全市有线数字电视整体转换工作。认真实施冠英街、滇南馆区域整体打造。全面开展第一次全国可移动文物普查。扎实推进文化遗产保护。

统筹抓好科技人才等社会事业。积极创建国家创新型城市，加快科技馆新馆建设，抓好高新技术企业、科技型中小企业发展和高新区创建工作。强力推进国家农业科技园区建设。进一步抓好国家知识产权试点城市工作。深入实施人才强市战略，创新人才选用渠道，用好用活现有人才。稳步实施生育政策的调整完善，继续推进幸福家庭创建活动，不断健全计生服务体系。广泛开展群众性体育活动，不断提高竞技体育水平，高质量承办四川省第十四届少数民族传统体育运动会，办好全市第四届老年人运动会。大力发展老龄事业，加快推进养老社区建设，完善养老服务体系。切实抓好统计工作，继续开展好第三次全国经济普查。努力争创全国质量强市示范城市，强化工商管理，提升食品药品安全保障水平。继续抓好地方志、爱国卫生、电子政务、住房公积金、科协、档案、工会、青少年、妇女儿童、民族宗教等事业发展。

加强和创新社会治理。加强基层社会治理和服务体系建设，充分发挥群众参与社会治理的基础作用，完善和创新流动人口和特殊人群管理服务。增强城乡社区服务功能，新建农村社区60个。深化“平安宜宾”建设，全面推进社会稳定风险评估，强化司法基本保障，依法防范和惩治违法犯罪活动。加强国家安全和保密工作。深入推进“六五”普法，进一步深化“法治宜宾”创建活动。畅通信访渠道，进一步办好“市长与网民”等平台。对口支援好甘孜州雅江县和新龙县经济社会发展。做好消防、地震、测绘、气象、水文工作，提高灾害防御能力。严格落实“一岗双责”，强力抓好安全生产，加大安全生产投入，健全应急管理体制机制，努力维护社会大局和谐稳定。

加强国防建设。推动军民融合深度发展，积极做好国防教育、国防动员、人民防空、交通战备等工作，进一步抓好征兵和民兵预备役建设。深入开展“全国双拥模范城” 创建活动，切实做好拥军优属和转业复员军人接收安置工作。

政府自身建设

各位代表，过去的一年，我们大力推进政府自身建设和管理创新，着力建设职能科学、结构优化、廉洁高效、人民满意的服务型政府，取得了较好成效。认真办理人大代表建议379件，政协委员提案313件，办复率为100%。着力转变政府职能，进一步优化市政府党组会、全体会、常务会等运行规则，强化政务调研和督查督办，推动发展的能力和水平不断提升。切实加强作风建设，各类文件和会议进一步减少，公务接待费用明显下降。深入推进行政审批制度改革和“两集中、两到位”工作，成为全省行政审批项目最少的市州之一，审批综合提速85.9%，名列全省前茅。全面推行公共资源交易电子化和政府信息公开标准化，政务环境进一步优化。大力推进依法治市，办理行政复议案件432件、法律援助4504件。加大监察、审计力度，反腐倡廉建设进一步加强。

我们清醒地认识到，政府自身建设与新形势新任务还不完全适应，一些地方科学发展意识不强，还存在形式主义、官僚主义等问题；一些干部宗旨意识淡薄，效率不高、作风不实，依法行政能力需要增强。我们将认真改进工作中存在的问题，积极参与以为民务实清廉为主要内容的党的群众路线教育实践活动，切实改革政府治理方式，以更加饱满的激情、更加务实的作风，勤勉尽责，开拓进取，忠实履行向全市人民作出的庄严承诺，向全市人民交上一份满意的答卷！

坚持放权简政，建设服务政府。认真开展行政体制改革，加快转变政府职能，切实接好中央和省上下放的审批事项，放好市本级该放的审批权力，加强事中事后监管，解放和增强社会活力。牢记全心全意为人民服务的宗旨，真正把以人为本、执政为民作为检验政府一切工作的最高标准。深入实施环境兴市战略，深化行政审批改革，抓好市政务中心和市公共资源交易中心建设，切实优化政务环境，提供优质公共服务。

坚持依法行政，建设法治政府。牢固树立依法治市理念，全面提高行政决策、执行、监督的法治化、民主化、科学化水平。在全社会形成办事依法、遇事找法、解决问题用法、化解矛盾靠法的环境，维护社会公众合法利益。强化行政监察、审计监督和司法监督，更加自觉地接受人大、政协的监督，更好地接受社会公众和媒体舆论监督。加强政务诚信建设，健全政务公开和各领域办事公开制度，让行政权力在阳光下运行。

坚持务实勤政，建设效能政府。切实加强领导班子建设，提升综合素质，贯彻好民主集中制，增强干事创业的整体功能。持续推进作风转变，将中央“八项规定”、省委和市委“十项规定”落实到政府工作的方方面面，切实做到踏石留印、抓铁有痕。大力精简会议、文件，转变会风、文风，提倡开短会、讲短话、行短文。大兴调查研究之风，积极开展“干部走基层、真情五为民”活动。进一步加强干部管理工作，围绕信念坚定、为民服务、勤政务实、敢于担当、清正廉洁的“好干部”标准，努力打造一支作风硬、素质高、能力强的公务员队伍。进一步健全绩效考评和行政问责制度，确保每一项决策都落到实处。

坚持从严治政，建设廉洁政府。全面落实党风廉政建设责任制，认真执行《建立健全惩治和预防腐败体系2013—2017年工作规划》，切实做到干部清正、政府清廉。加强对重要岗位、关键环节的监督和制约，强化领导干部经济责任审计，规范权力运行。进一步规范工程建设招投标、土地使用权和矿业权出让、国有产权交易、政府采购等工作。全面贯彻《机关事务管理条例》，坚决执行《党政机关厉行节约反对浪费条例》，严格控制评比、达标、表彰活动和公费出国等经费支出。

各位代表，面对全市人民的信任和重托，面对新的挑战和考验，我们倍感使命在肩、责任重大。让我们紧密地团结在以习近平同志为总书记的党中央周围，在省委、省政府和市委的坚强领导下，拼搏实干、克难攻坚，稳位求效、创新求进，为实现率先崛起、全面建成小康的宏伟目标而努力奋斗！
